# Supplementary figures and images for: Epidemiology and burden of focal segmental glomerulosclerosis among United States Veterans: An analysis of Veteran’s Affairs data
Source: PLoS One. 2024 Dec 13;19(12):e0315302. doi: 10.1371/journal.pone.0315302 (PMC11642916; doi:10.1371/journal.pone.0315302)

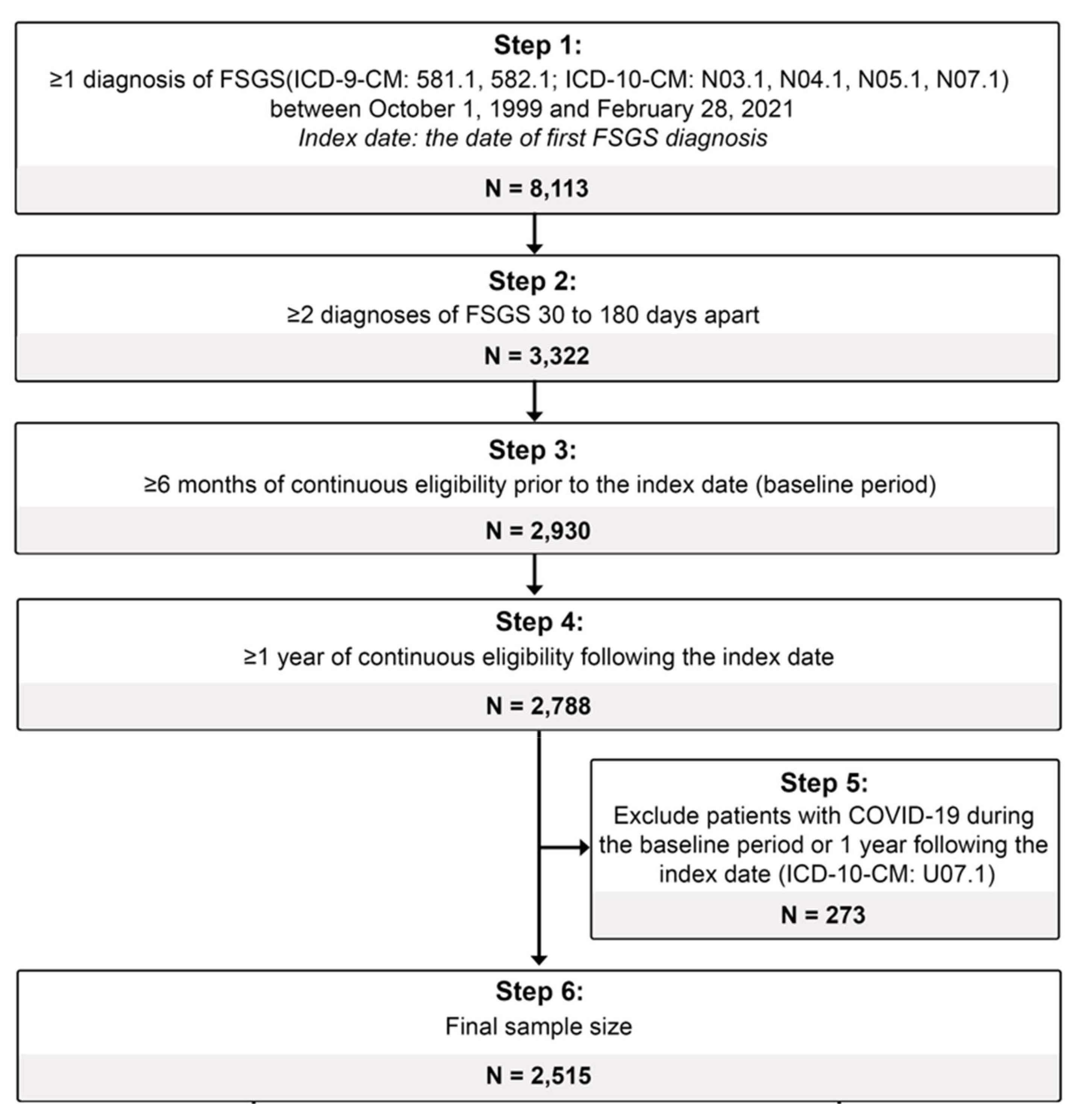

Supplement: S1 Fig — Data Source: Veteran Affairs Health System Corporate Data Warehouse (October 1999—February 2021). Abbreviations: COVID-19, coronavirus 2019; FSGS, focal segmental glomerulosclerosis; ICD-9/10- CM, International Classification of Diseases, Ninth/Tenth Revision, Clinical Modification. (TIF) [file pone.0315302.s001.tif]
