# Supplementary material for: Epidemiology and burden of focal segmental glomerulosclerosis among United States Veterans: An analysis of Veteran’s Affairs data
Source: PLoS One. 2024 Dec 13;19(12):e0315302. doi: 10.1371/journal.pone.0315302 (PMC11642916; doi:10.1371/journal.pone.0315302)
Supplement: S1 Table — Abbreviations: ACE, angiotensin-converting enzyme; FSGS, focal segmental glomerulosclerosis; MRA, mineralocorticoid receptor antagonist; SD, standard deviation; SGLT2, sodium-glucose transport protein 2. Notes: a The follow-up period spanned from the index date (the date of first diagnosis date of FSGS) to the end of enrollment date, end of data availability, or date of death, whichever occurred first. b Among patients who had ≥2 years or ≥3 years of continuous eligibility following the index date. (DOCX) [file pone.0315302.s002.docx]

**S1 Table. Medication used during the follow-up period,a by drug class: United States, 2000–2020**

|  | **1^st^ Year** | **2^nd^ Year^b^** | **3^rd^ Year^b^** | **Follow-up Period^a^** |
| --- | --- | --- | --- | --- |
|  | **N = 2,515** | **N = 2,344** | **N = 2,152** | **N = 2,515** |
| **Medication use, n (%)**  ACE inhibitors | 1,506 (59.9) | 1,191 (50.8) | 983 (45.7) | 1,757 (69.9) |
| Number of prescriptions per patient, mean (SD) [median] | 4.3 (2.7) [4.0] | 3.9 (2.3) [4.0] | 3.8 (2.4) [4.0] | 19.9 (19.1) [14.0] |
| Angiotensin receptor blocker | 338 (13.4) | 285 (12.2) | 228 (10.6) | 548 (21.8) |
| Number of prescriptions per patient, mean (SD) [median] | 4.3 (2.7) [4.0] | 3.8 (2.5) [4.0] | 4.0 (2.5) [4.0] | 15.0 (17.7) [9.0] |
| Beta blockers | 707 (28.1) | 626 (26.7) | 563 (26.2) | 1,363 (54.2) |
| Number of prescriptions per patient, mean (SD) [median] | 4.1 (2.3) [4.0] | 3.9 (2.2) [4.0] | 3.8 (2.1) [4.0] | 16.2 (15.8) [11.0] |
| Calcineurin inhibitor | 285 (11.3) | 211 (9.0) | 178 (8.3) | 542 (21.6) |
| Number of prescriptions per patient, mean (SD) [median] | 6.0 (4.8) [5.0] | 6.0 (4.2) [5.0] | 5.4 (4.1) [4.0] | 22.9 (28.2) [13.0] |
| Calcium channel blockers | 1,364 (54.2) | 1,150 (49.1) | 1,023 (47.5) | 1,889 (75.1) |
| Number of prescriptions per patient, mean (SD) [median] | 4.3 (2.5) [4.0] | 4.3 (2.5) [4.0] | 4.0 (2.2) [4.0] | 21.7 (19.4) [16.0] |
| Diuretics | 846 (33.6) | 632 (27.0) | 503 (23.4) | 1,340 (53.3) |
| Number of prescriptions per patient, mean (SD) [median] | 4.2 (3.1) [4.0] | 3.8 (2.7) [3.0] | 3.7 (2.3) [3.0] | 13.9 (15.7) [8.0] |
| Glucagon-like peptide 1 receptor agonist | 3 (0.1) | 4 (0.2) | 6 (0.3) | 62 (2.5) |
| Number of prescriptions per patient, mean (SD) [median] | 6.7 (6.0) [6.0] | 5.3 (2.8) [5.5] | 5.2 (2.9) [4.5] | 12.5 (12.5) [8.5] |
| Glucocorticoids | 1,049 (41.7) | 676 (28.8) | 549 (25.5) | 1,762 (70.1) |
| Number of prescriptions per patient, mean (SD) [median] | 4.4 (3.4) [4.0] | 3.8 (3.0) [3.0] | 3.7 (3.0) [3.0] | 13.6 (19.0) [6.0] |
| Mineralocorticoid receptor antagonist | 200 (8.0) | 139 (5.9) | 120 (5.6) | 413 (16.4) |
| Number of prescriptions per patient, mean (SD) [median] | 3.5 (2.5) [3.0] | 3.5 (2.4) [3.0] | 3.8 (2.8) [3.0] | 9.2 (11.7) [5.0] |
| Potassium binders | 134 (5.3) | 106 (4.5) | 95 (4.4) | 490 (19.5) |
| Number of prescriptions per patient, mean (SD) [median] | 2.3 (1.9) [2.0] | 2.2 (2.1) [1.0] | 2.2 (2.2) [1.0] | 4.4 (8.0) [2.0] |
| SGLT2 inhibitor | 7 (0.3) | 8 (0.3) | 7 (0.3) | 78 (3.1) |
| Number of prescriptions per patient, mean (SD) [median] | 3.1 (1.1) [3.0] | 5.3 (4.2) [4.0] | 2.3 (1.0) [3.0] | 6.0 (5.1) [4.0] |
| Statins | 1,658 (65.9) | 1,472 (62.8) | 1,322 (61.4) | 2,061 (81.9) |
| Number of prescriptions per patient, mean (SD) [median] | 4.1 (2.4) [4.0] | 3.9 (2.2) [4.0] | 3.8 (2.1) [4.0] | 24.6 (22.0) [18.0] |

**Abbreviations**: ACE, angiotensin-converting enzyme; FSGS, focal segmental glomerulosclerosis; MRA, mineralocorticoid receptor antagonist; SD, standard deviation; SGLT2, sodium-glucose transport protein 2. **Notes:** ^a^ The follow-up period spanned from the index date (the date of first diagnosis date of FSGS) to the end of enrollment date, end of data availability, or date of death, whichever occurred first. ^b^ Among patients who had ≥2 years or ≥3 years of continuous eligibility following the index date.
